# Peak knee joint moments accurately predict medial and lateral knee contact forces in patients with valgus malalignment

**DOI:** 10.1038/s41598-023-30058-4

**Published:** 2023-02-18

**Authors:** Jana Holder, Stefan van Drongelen, Scott David Uhlrich, Eva Herrmann, Andrea Meurer, Felix Stief

**Affiliations:** 1 Movement Analysis Laboratory, Department of Orthopedics (Friedrichsheim), University Hospital Frankfurt, Goethe University Frankfurt, Frankfurt/Main, Germany; 2 Present Address: Department of Sport and Exercise Science, University of Salzburg, Salzburg, Austria; 3 Dr. Rolf M. Schwiete Research Unit for Osteoarthritis, Department of Orthopedics (Friedrichsheim), University Hospital Frankfurt, Goethe University Frankfurt, Frankfurt/Main, Germany; 4 Department of Bioengineering, Stanford University, Stanford, CA USA; 5 Musculoskeletal Research Lab, VA Palo Alto Healthcare System, Palo Alto, CA USA; 6 Institute of Biostatistics and Mathematical Modeling, Goethe University Frankfurt, Frankfurt/Main, Germany; 7 Department of Orthopedics (Friedrichsheim), University Hospital Frankfurt, Goethe University Frankfurt, Frankfurt/Main, Germany; 8 Present Address: Medical Park St. Hubertus Klinik, Bad Wiessee, Germany

**Keywords:** Paediatric research, Biomedical engineering

## Abstract

Compressive knee joint contact force during walking is thought to be related to initiation and progression of knee osteoarthritis. However, joint loading is often evaluated with surrogate measures, like the external knee adduction moment, due to the complexity of computing joint contact forces. Statistical models have shown promising correlations between medial knee joint contact forces and knee adduction moments in particularly in individuals with knee osteoarthritis or after total knee replacements (*R*^2^ = 0.44–0.60). The purpose of this study was to evaluate how accurately model-based predictions of peak medial and lateral knee joint contact forces during walking could be estimated by linear mixed-effects models including joint moments for children and adolescents with and without valgus malalignment. Peak knee joint moments were strongly correlated (*R*^2^ > 0.85, *p* < 0.001) with both peak medial and lateral knee joint contact forces. The knee flexion and adduction moments were significant covariates in the models, strengthening the understanding of the statistical relationship between both moments and medial and lateral knee joint contact forces. In the future, these models could be used to evaluate peak knee joint contact forces from musculoskeletal simulations using peak joint moments from motion capture software, obviating the need for time-consuming musculoskeletal simulations.

## Introduction

In the last five years the number of studies has tripled (see supplementary material for the full search terms used in Pubmed) that performed sports or clinical gait analysis and investigated internal joint contact or muscle forces rather than joint moments^1^. The knee adduction moment (KAM) is a commonly used surrogate measure for medial compartment knee loading because it was statistically associated to osteoarthritis (OA) severity and progression^2,3^ and is relatively simple to compute. Calculating joint contact forces require the additional use of musculoskeletal simulation software. They are a part of the internal load and mainly generated by muscles during walking^4,5^. Both methods for estimating joint loading demonstrate advantages: joint moments are easily calculated, but knee joint contact forces are more representative of cartilage loading^6^. Joint moments are usually available almost directly after the movement analysis because joint moment output calculated by inverse dynamics is often implemented in the standard data acquisition software^7^. The external knee flexion/extension moment (KFM/KEM) also contributes to the internal knee joint contact force (KCF). Linear models that use both the KAM and KFM as covariates have higher correlations with KCF than models that use KAM alone^8–10^. Calculating the internal joint contact, muscle and/or tendon forces require additional time and expertise^1,11,12^. Therefore, the calculation of joint moments has the advantage of quick availability and lower cost in terms of time or human capacity. Estimating internal joint contact forces with musculoskeletal models include the contribution of all internal forces such as muscles and ligaments (when present in the model). Hence, internal joint contact forces provide a more accurate measure of joint loading compared to joint moments during dynamic tasks as walking. Nevertheless, both methods are estimations of the loading in a joint^13^. In vivo measurement of joint contact force can only be done by invasive methods as an instrumented prosthesis^14^. Patients with instrumented prostheses are rare^15,16^, and their loading patterns may not be representative of other populations of interest, like children. Additionally, highly dynamic movements like side-cutting have not been investigated in patients with instrumented prosthesis. The other two named methods, calculating external joint moments and internal joint contact forces, are therefore used in a more dynamic environment when younger study cohorts and other dynamic movements except for walking are investigated.

In a clinical setting, methods for estimating joint loading that are both accurate and inexpensive are needed, e.g. in young patients with a static frontal plane deformity at the knee joint as valgus or varus due to an increased risk of developing knee OA^17^. Young patients with a pathological leg alignment e.g. valgus, and remaining growth potential can be treated by guided growth^18^. During this treatment the pathological leg alignment is changed over time with the aim to reduce the lateral knee joint loading. Especially in borderline cases, when the measured parameters from a static X-ray image does not reveal a clear medical indication for or against the guided growth intervention, dynamic loading parameters from gait analysis are additionally assessed^19^. Previous studies showed that with a valgus malalignment, the external KAM and medial knee joint contact force (medKCF) during walking are reduced whereas the lateral knee joint contact force (latKCF) is increased^20–22^. Similar results were found in patients with medial knee OA who walk with an increased KAM^23^ and a larger medKCF compared to age-matched healthy controls^24^. The KAM and medKCF correlate moderate to good during first (*R*^2^ = 0.45–0.60) and second half of stance (*R*^2^ = 0.44–0.55)^25–27^ in patients with medial knee OA or after total knee replacement. In general, the relationship between knee joint moments (KJMs) and latKCF has been less studied^28–31^, and most cohorts are older adults or individuals with knee OA^25–27^. The ability of joint moments to predict medKCF in these cohorts is promising, but further work is needed to understand these relationships in young individuals with valgus malalignment who are at increased risk of developing OA.

The aim of this study was to develop statistical models that relate peak external knee joint moments (i.e., the knee adduction and flexion moments) to internal knee joint contact forces (i.e., medial and lateral knee joint contact force peaks) in the first and second half of stance, during walking in young patients with and without valgus malalignment. We hypothesized that 1) the predictive accuracy of statistical models that estimate knee joint contact force peaks in children and adolescents with and without valgus malalignment from peak external joint moments is high^26,32^ (*R*^2^ > 0.49; RMSE < 10%); 2) the predictive accuracy is larger for the medial knee joint contact force compared to the lateral knee joint contact force; and 3) the predictive accuracy of the statistical models that use both sagittal and frontal plane moments to predict the knee joint contact forces will be greater than those that use joint moments from a single plane.

## Results

### Anthropometrics and walking speed

For comparing the anthropometrics and walking speed between groups, we performed independent *t*-tests or Mann–Whitney-*U*-test for not normally distributed data. These results are summarized in Table 1. All parameters except for age were normally distributed. The study groups were significantly different in body height (*p* = 0.014), body mass (*p* < 0.001), body mass index (*p* < 0.001) and the mechanical axis angle (*p* < 0.001) but not for age and walking speed (*p* > 0.05). The effect sizes were large for all parameters except for age, body height and walking speed.

**Table 1:** Anthropometrics and walking speed.

	Patient group	Shapiro–Wilk (*p*-value)	TD group	Shapiro–Wilk (*p*-value)	Comparison between groups	
					*t*-test / Mann–Whitney-*U*-test (*p*-value)	Effect size* r*
Number of participants	50		21			
Sex [female / male]	19 / 31		7/14			
Bilateral / left / right affected limbs	38 / 5 / 7		–/10/11			
Age [years]	13.0 (11.0–13.0)	< 0.001	12.0 (12.0–14.0)	0.004	0.294*	0.124
Body height [m]	1.66 ± 0.10	0.379	1.59 ± 0.10	0.612	0.014	0.291
Body mass [kg]	63.5 ± 13.7	0.680	46.1 ± 10.7	0.364	< 0.001	0.530
Body mass index [kg/m^2^]	23.0 ± 3.4	0.204	18.1 ± 2.5	0.132	< 0.001	0.585
Mechanical axis angle [°]	− 6.0 ± 1.8	0.248	-0.0 ± 2.3	0.379	< 0.001	0.811
Walking speed [m/s]	1.25 ± 0.16	0.569	1.29 ± 0.17	0.810	0.368	0.109

TD: Typically developed healthy control group; Mechanical axis angle of the patients was measured by an X-ray image; mechanical axis angle of the TD group was measured from the static trial from the three-dimensional gait analysis; Normal distributed data displayed as mean ± standard deviation; not normally distributed data are displayed as median (25. quartile—75. quartile) and marked with a *; Mann–Whitney-*U*-tests have been performed instead of independent *t*-tests for not normally distributed data; significant *p*-values are highlighted in bold; Effect size *r* > 0.1: small; *r* > 0.3: medium; *r* > 0.5: strong.

### Gait kinematics and kinetics

**Figure 1:**
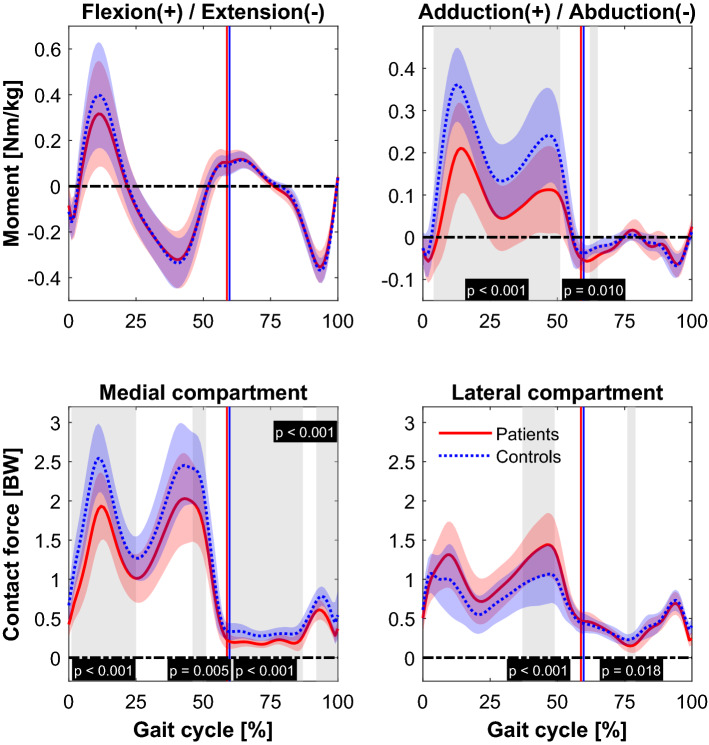
The mean (line) and standard deviation (shaded) of the external knee flexion and adduction moment and the medial and lateral knee contact force of the patients with valgus malalignment (red, solid) and the typically developed healthy controls (blue, dashed) are displayed. Vertical lines mark the end of the stance phase. Joint moments were normalized by body mass (unit: Nm/kg) and the joint contact forces by body weight (unit: BW). Significant different phases (*p* < 0.05) during the gait cycle (normalized to 100%) calculated with a statistical parametric mapping two sample *t*-test are highlighted with gray areas and are described with the associated *p*-value (black boxes).

The mean curves of the dynamic KJMs and KCFs were compared between the two groups using statistical parametric mapping (Fig. 1). The KFM did not significantly differ between the patient and typically developed healthy control (TD) group. KAM was significantly smaller in the patient group between 3 and 52% (*p* < 0.001) and 61–66% (*p* = 0.010) of the gait cycle. The medKCF was significantly smaller in the patient group between 0 and 25% (*p* < 0.001), between 46 and 52% (*p* = 0.005), between 58 and 88% (*p* < 0.001), and between 91 and 100% (*p* < 0.001). The latKCF was significantly increased for the patient group between 37 and 50% (*p* < 0.001) and between 75 and 80% (*p* = 0.018) of the gait cycle. Other kinematic and kinetic curves and comparisons are included in the supplementary material.

### Linear models

To establish the relationships between single-plane KJMs and KCFs predicted via musculoskeletal models, we first investigated correlations of KAM or KFM and predicted medKCF or latKCF for the peaks in the first and second half of stance individually. Low to moderate correlations of *R*^2^ < 0.49 were detected except between KAM2 and latKCF2 (*R*^2^ = 0.68) for the patient group and KAM2 and medKCF2 (*R*^2^ = 0.59) for the TD group. The root mean squared error (*RMSE*) ranged between 14 and 29%. See full results in the supplementary material, Tables 1 and 2.

**Table 2:** Statistical summary of the linear mixed-effects models between the internal knee joint contact forces and external knee joint moments for the patient group.

Response Variable	Predictor Variable	Estimate	Standard Error	*t*-value	Degrees of freedom	*p*-value	Lower 95% CI	Upper 95% CI	Adj *R*^2^	*RMSE*	*RMSE* [%]
medKCF1	Intercept	1.411	0.049	29.009	417	< 0.001	1.315	1.506	0.90	0.14	7.01
	KAM1	2.187	0.166	13.206	417	< 0.001	1.861	2.513			
	qKFM1	0.551	0.073	7.513	417	< 0.001	0.407	0.695			
medKCF2	Intercept	1.202	0.054	22.272	417	< 0.001	1.096	1.308	0.96	0.13	6.0
	KAM2	3.012	0.228	13.184	417	< 0.001	2.563	3.462			
	qKEM2	4.158	0.209	19.863	417	< 0.001	3.747	4.570			
latKCF1	Intercept	1.544	0.044	35.012	417	< 0.001	1.457	1.630	0.89	0.15	10.3
	KAM1	− 1.559	0.162	− 9.649	417	< 0.001	− 1.876	− 1.241			
	qKFM1	1.498	0.117	12.819	417	< 0.001	1.269	1.728			
latKCF2	Intercept	1.754	0.042	41.818	417	< 0.001	1.671	1.836	0.95	0.10	6.8
	KAM2	− 2.176	0.135	− 16.146	417	< 0.001	− 2.441	− 1.911			
	qKEM2	0.454	0.141	3.218	417	0.001	0.177	0.731			

CI: Confidence interval; Adj. *R*^2^: adjusted *R*^2^*; RMSE*: root mean squared error [BW]; medKCF1/medKCF2: max. value in the first/second half of stance of the medial knee joint contact force [BW]; latKCF1/latKCF2: max. value in the first/second half of stance of the lateral knee joint contact force [BW]; KAM1/KAM2: max. value in the first/second half of stance of the external knee adduction moment [Nm/kg]; qKFM1/qKEM2: squared maximal/minimal value in the first/second half of stance of the external knee flexion/extension moment (KFM1, KEM2; [Nm/kg]).

### Linear mixed-effects models

For testing the possibility of accurately predicting peaks of medKCF and latKCF by combining KAM and KFM/KEM, we used linear mixed-effects models (LMM). For improvement of the model, random effects for both included limbs from bilaterally affected patients and different numbers of included trials per participant were added.

#### Patients

Equations (1) to (4) in the Supplementary Material describe the LMMs that relate knee moments to the first and second peaks of medKCF and latKCF in the patient group. The first and second peaks of the variables are denoted by appending the peak number to the end of the variable (e.g., the first peak KAM is KAM1). The results of the LMMs are summarized in Table 2. For all four LMMs, KAM, and the squared knee flexion/extension moment (qKFM1, qKEM2) were included as significant fixed and random effects. All four LMMs reported an adjusted *R*^2^ between 0.89 and 0.96.

#### Typically developed healthy controls

For the TD group LMMs were also performed for the four parameters medKCF1, medKCF2, latKCF1 and latKCF2 and shown in Eqs. (5) to (8) in the Supplementary Material. Similar *R*^2^ values were found (between 0.92 and 0.97) compared to the results from the patient group (Table 3).

**Table 3:** Statistical summary of the linear mixed-effects models between the internal knee joint contact forces and external knee joint moments for the typically developed healthy control group.

Response Variable	Predictor Variables	Estimate	Standard Error	*t*-value	Degrees of Freedom	*p*-value	Lower 95% CI	Upper 95% CI	Adj *R*^2^	*RMSE*	*RMSE* [%]
medKCF1	Intercept	1.480	0.138	10.740	93	< 0.001	1.207	1.754	0.95	0.12	4.9
	KAM1	2.423	0.438	5.525	93	< 0.001	1.552	3.294			
	qKFM1	0.777	0.130	5.956	93	< 0.001	0.518	1.036			
medKCF2	Intercept	1.289	0.123	10.495	93	< 0.001	1.045	1.533	0.97	0.13	4.7
	KAM2	2.902	0.485	5.984	93	< 0.001	1.939	3.865			
	qKEM2	4.133	0.483	8.550	93	< 0.001	3.173	5.093			
latKCF1	Intercept	1.390	0.156	8.936	93	< 0.001	1.081	1.699	0.92	0.12	10.2
	KAM1	− 1.112	0.444	− 2.502	93	0.014	− 1.995	− 0.230			
	qKFM1	0.976	0.141	6.904	93	< 0.001	0.695	1.257			
latKCF2	Intercept	1.726	0.094	18.372	94	< 0.001	1.539	1.912	0.92	0.11	9.5
	KAM2	-2.160	0.279	-7.745	94	< 0.001	− 2.714	− 1.607			

CI: Confidence interval; Adj. *R*^2^: adjusted *R*^2^*; RMSE*: root mean squared error [BW]; medKCF1/medKCF2: max. value in the first/second half of stance of the medial knee joint contact force [BW]; latKCF1/latKCF2: max. value in the first/second half of stance of the lateral knee joint contact force [BW]; KAM1/KAM2: max. value in the first/second half of stance of the external knee adduction moment [Nm/kg]; qKFM1/qKEM2: squared maximal/minimal value in the first/second half of stance of the external knee flexion/extension moment (KFM1, KEM2; [Nm/kg]).

Since the outcome and the structure of the LMMs were comparable, the datasets of both groups were combined for estimating LMMs independent of the studied group. To consider possible effects of the different groups, a categorical variable as fixed effect was included when building the LMMs for the combined dataset. The equations and results from these models are displayed in the following Eqs. (1–4) and Table 4. Comparable LMMs with *R*^2^ between 0.88 and 0.96 were found as for the studied groups individually (medKCF1: *R*^2^ = 0.91 (Eq. 1); medKCF2: *R*^2^ = 0.96 (Eq. 2); latKCF1: *R*^2^ = 0.88 (Eq. 3); latKCF2: *R*^2^ = 0.94 (Eq. 4)).

**Table 4:** Statistical summary of the linear mixed-effects models between the internal knee joint contact forces and external knee joint moments for the complete dataset (patient and typically developed healthy control group).

Response Variable	Predictor Variables	Estimate	Standard Error	*t*-value	Degrees of Freedom	*p*-value	Lower 95% CI	Upper 95% CI	Adj *R*^2^	*RMSE*	*RMSE* [%]
medKCF1	Intercept	1.447	0.055	26.494	512	< 0.001	1.340	1.555	0.92	0.15	7.0
	KAM1	2.003	0.198	10.098	512	< 0.001	1.613	2.392			
	qKFM1	0.576	0.060	9.605	512	< 0.001	0.458	0.694			
	KAM1:groupVar	0.404	0.095	4.243	512	< 0.001	0.217	0.591			
medKCF2	Intercept	1.180	0.054	21.970	513	< 0.001	1.074	1.285	0.96	0.14	6.3
	KAM2	3.095	0.244	12.692	513	< 0.001	2.616	3.574			
	qKEM2	4.201	0.194	21.654	513	< 0.001	3.819	4.582			
latKCF1	Intercept	1.519	0.059	25.862	510	< 0.001	1.404	1.635	0.88	0.15	10.9
	KAM1	− 1.507	0.195	− 7.728	510	< 0.001	− 1.890	− 1.124			
	qKFM1	1.600	0.129	12.453	510	< 0.001	1.348	1.853			
	groupVar	− 0.309	0.112	-2.773	510	0.006	− 0.528	− 0.090			
	KAM1:groupVar	1.018	0.371	2.742	510	0.006	0.288	1.747			
	KAM1:qKFM1:groupVar	− 2.291	0.392	-5.838	510	< 0.001	− 3.062	− 1.520			
latKCF2	Intercept	1.802	0.044	41.022	512	< 0.001	1.716	1.888	0.94	0.11	7.8
	KAM2	− 2.124	0.160	− 13.274	512	< 0.001	− 2.438	-1.810			
	groupVar	− 0.088	0.031	-2.874	512	0.004	− 0.149	-0.028			
	KAM2:qKEM2:groupVar	− 1.128	0.817	− 1.380	512	0.168	− 2.733	0.478			

CI: Confidence interval; Adj. *R*^2^: adjusted *R*^2^*; RMSE*: root mean squared error [BW]; medKCF1/medKCF2: max. value in the first/second half of stance of the medial knee joint contact force [BW]; latKCF1/latKCF2: max. value in the first/second half of stance of the lateral knee joint contact force [BW]; KAM1/KAM2: max. value in the first/second half of stance of the external knee adduction moment [Nm/kg]; qKFM1/qKEM2: squared maximal/minimal value in the first/second half of stance of the external knee flexion/extension moment (KFM1, KEM2; [Nm/kg]).

1 $$\mathrm{medKCF}1=1.447+2.003\times\mathrm{KAM}1+0.576\times\mathrm{qKFM}1+0.404\times\mathrm{KAM}1:\mathrm{groupVar}+\left(1+\mathrm{KAM}1+\mathrm{qKFM}1|\mathrm{subjVar}\right)+\left(1|\mathrm{subjVar}:\mathrm{footVar}\right)$$

2 $$\mathrm{medKCF}2=1.180+3.095\times \mathrm{KAM}2+4.201\times \mathrm{qKEM}2+(1+\mathrm{KAM}2+\mathrm{qKEM}2|\mathrm{subjVar})+(1|\mathrm{subjVar}:\mathrm{footVar})$$

3 $$\mathrm{latKCF}1=1.519+\left(-1.507\right)\times \mathrm{KAM}1+1.600\times \mathrm{qKFM}1+\left(-0.309\right)\times \mathrm{groupVar}+1.018\times \mathrm{KAM}1:\mathrm{groupVar}+\left(-2.291\right)\times \mathrm{KAM}1:\mathrm{qKFM}1:\mathrm{groupVar}+(1+\mathrm{KAM}1+\mathrm{qKFM}1|\mathrm{subjVar})+(1|\mathrm{subjVar}:\mathrm{footVar})$$

4 $$\mathrm{latKCF}2=1.802+\left(-2.124\right)\times \mathrm{KAM}2+\left(-0.088\right)\times \mathrm{groupVar}+\left(-1.128\right)\times \mathrm{KAM}2:\mathrm{qKEM}2:\mathrm{groupVar}+(1+\mathrm{KAM}2+\mathrm{qKEM}2|\mathrm{subjVar})+(1|\mathrm{subjVar}:\mathrm{footVar})$$

Leave-one-out cross validation of the LMMs from the combined dataset revealed high accuracy (*R*^2^ between 0.83 and 0.93) in the prediction of the peak values of medKCF and latKCF with the peak knee joint moments from the sagittal and frontal plane (Table 5).

**Table 5:** Statistical summary of the leave one out cross-validation of the linear mixed-effects models between the internal knee joint contact forces and external knee joint moments for the complete dataset (patient and typically developed healthy control group).

Response	Linear mixed-effects model	*RMSE*	*SSE*	*SST*	*SSR*	*R*^2^
medKCF1	1 + KAM1 + qKFM1 + KAM1:groupVar	0.17	14.98	115.91	102.14	0.88
medKCF2	1 + KAM2 + qKEM2	0.17	15.08	201.61	188.00	0.93
latKCF1	1 + KAM1 + qKFM1 + groupVar + KAM1:groupVar + KAM1:qKFM1:groupVar	0.18	17.15	82.60	68.26	0.83
latKCF2	1 + KAM2 + groupVar + KAM2:qKEM2:groupVar	0.13	8.54	85.37	76.95	0.90

medKCF1/medKCF2: max. value in the first/second half of stance of the medial knee joint contact force [BW]; latKCF1/latKCF2: max. value in the first/second half of stance of the lateral knee joint contact force [BW]; KAM1/KAM2: max. value in the first/second half of stance of the external knee adduction moment [Nm/kg]; qKFM1/qKEM2: squared maximal/minimal value in the first/second half of stance of the external knee flexion/extension moment (KFM1, KEM2; [Nm/kg]); *RMSE*: root mean squared error [BW]; *SST:* sum of squares total; *SSR*: sum of squares regression; *SSE*: sum of squares error.

## Discussion

We investigated the accuracy of statistical models that predict peak internal knee joint contact forces from peak knee joint moments in children and adolescents with and without valgus malalignment. We found that the linear mixed-effects models could predict medial and lateral knee contact force peaks with a high accuracy of *R*^2^ > 0.87 and *RMSE* < 16% when including knee joint moments from both the sagittal and frontal plane. The first hypothesis was confirmed that the peak knee contact forces can be predicted with high accuracy with linear mixed-effects models. The second hypothesis was rejected, because both peaks of medial and lateral knee contact forces, could be predicted with high accuracy (*R*^2^ > 0.87) from peak joint moments. Our third hypothesis was confirmed, as models that included knee moments in the sagittal and frontal plane predicted joint contact forces with higher accuracy (*R*^2^ = 0.88–0.96) than those that used a moment in a single plane (*R*^2^ = 0.01–0.68). These results suggest that peak internal knee contact forces from musculoskeletal simulations calculated with static optimization can be accurately predicted with statistical linear mixed-effects models that use inputs from commonly-used gait analysis tools. There linear equations mitigate the need for complex musculoskeletal modeling procedures and potentially enabling these estimates to be made in a clinical setting when evaluating children and adolescents with valgus leg alignment. The leg alignment of these children and adolescents can be changed by guided growth. The deviation or no deviation of the medial and lateral knee joint contact force from a predefined normative area could either confirm or disconfirm the medical indication for a guided growth. With the linear equations from the linear mixed-effects models the peak medial or lateral knee contact forces are available almost immediately after a gait analysis and time consuming musculoskeletal simulations do not need to be performed.

Previous studies found that KAM correlated well with medKCF in the first half of stance with a prediction accuracy of about *R*^2^ ≈ 0.4 and were performed in patients with knee OA or after knee replacement^25,27,29^. Previous studies also investigated the relationship between KFM and medKCF with, in general, low correlations (*R*^2^ < 0.25) for both peaks^24,29,33,34^. Moreover, in few studies, multivariate models were used to study the effect of KAM and KFM on medKCF. These studies improved the prediction of medKCF for the first peak (*R*^2^ improved by approximately 0.2) but not for the second peak. In general, the reported *R*^2^ for the first peak of medKCF using KAM and KFM varied between 0.54 and 0.85 in older adults with and without musculoskeletal pathologies^9,10,26^. The lateral knee joint contact force has been less investigated and the few studies performed only found low relations with KAM (*R*^2^ < 0.15) and slightly stronger correlations with KFM (*R*^2^ < 0.3)^28–30^. In the present study, the LMMs revealed large predictive power of *R*^2^ > *0.88* and a *RMSE* ≤ 10.9%. These results on the one hand strengthen the possibility of an accurate determination of peak internal KCFs from musculoskeletal simulations by peak external KAM and KFM/KEM for young individuals with and without valgus malalignment. On the other hand, our results in combination with the literature reveal that a high correlation between knee joint moments and joint contact forces also depend on the study cohort.

Studies found that reducing KAM with gait modifications does not necessarily also change medKCF because other joint loading parameters as KFM or muscle co-contraction might be affected^10,35–37^. A possible successful gait modification for reducing medKCF could be in-toeing that potentially reduces KAM but not substantially affecting KFM^38–40^. The effect of gait modifications on latKCF has not been investigated in the past. Previous studies found KFM or KEM as the main contributor to latKCF^41^, suggesting that offloading gait should target these parameters.^36,39^ However, this presumption need to be validated in an experimental study. The two LMMs from the present study revealed a negatively directed correlation between KAM and latKCF. This suggests that an increase of KAM and no change of KFM could reduce latKCF. Nonetheless, gait modifications often alter both KAM and KFM and affect both medKCF and latKCF; though, the relative contribution of the moments differ between compartments and peak times. Future studies should use models, like the ones presented here, that consider the effects of both KAM and KFM on KCF.

Most other studies relating KJMs to KCFs investigate older populations with knee OA who likely have varus or neutral frontal plane alignment^25^. Our results provide estimates of loading in young patients with valgus malalignment that may inform the need for guided growth intervention in these children and adolescents. Currently, the decision for a guided growth intervention is based on the static mechanical axis angle from an X-ray image, which is not highly correlated with medKCF and latKCF^22^. Consequently, this study may be helpful in the decision-making for guided growth. Moreover, the coefficients of our models are mostly different from those in models that used patients with varus alignment. In our models, the extracted values of KFM/KEM were included as squared parameters because a high variance was found. This highlights the importance of using population-specific models to determine internal KCFs.^24,42,43^ Moreover, the more complicated LMMs for predicting the peak latKCF especially in the first half of stance but also in the second half strengthen the understanding, that the prediction of latKCF is dependent on both KAM and KFM/KEM.

## Limitations

It is important to identify the limitations of the study. This study demonstrates that peak KCFs can be accurately predicted using peak KJMs from OpenSim; however, a more clinically applicable solution would be to use the peak KJMs directly from the three-dimensional motion capture system. We used KJMs from OpenSim to avoid confounding effects of differing coordinate systems, which can influence kinematic and kinetic results^44,45^. Future studies should investigate the influence of different coordinate systems or models on the relationship between joint moments and joint contact forces. Alternatively, a transformation between the motion capture and OpenSim coordinate systems could be determined and applied to the joint moment data prior to using our model. Additionally, with these LMMs only the peak KCFs can be predicted and only a limited indication is possible for the entire gait cycle. Furthermore, the hip joint centers were calculated based on the definitions from Davis, et al.^7^ and could be improved in future studies. Moreover, the musculoskeletal models were linearly scaled based on marker positions but not reconstructed from participant-specific medical images because only two-dimensional full leg X-ray images were available for the patient group. Linearly scaled models might affect joint center definitions, joint moment and muscle moment arm calculations^46–48^. However, apart from the pathological leg axis in the frontal plane, which was implemented in the participant-specific models, no other pathological anatomies were diagnosed in our study participants. Medical examinations and measurements of the passive range of motion excluded malalignments, e.g. in the transverse plane such as increased antetorsion. Moreover, we used a cost function that minimized the sum of squared muscle activation in the static optimization approach to model the muscle activation and forces and did not include participant-specific surface electromyography data. Therefore, our simulations lacked in the ability of representing participant-specific activation patterns^37^. Lastly, for fulfilling a complete view of leg alignment in children and adolescents and the influence of leg alignment on internal joint contact force, patients with varus alignment should be included. Although, in our hospital children and adolescents with varus alignment are also part of a large study, the number of participants is still small and therefore were not included.

## Conclusion

We investigated the relationship between knee joint moments and knee joint contact forces in children and adolescents with and without valgus malalignment during walking. The predictions from linear mixed-effects models were strongly related to knee joint contact force peaks from musculoskeletal simulations. This suggests that knee joint contact forces could be estimated in the future using knee joint moments from standard motion capture software as input to the linear mixed-effects models. Furthermore, including both the knee flexion/extension and adduction moments in the linear mixed-effects models increased the prediction accuracy. This supports the importance of evaluating the role of both muscle forces and dynamic mechanics in medial and lateral knee joint contact forces. By simplifying the evaluation of internal joint loading, the statistical models may enable clinicians and researchers to study and prescribe gait modifications that reduce knee joint contact force without needing to perform time-consuming musculoskeletal simulations.

## Methods and materials

### Participants

In total, 71 children and adolescents were included in this study, 50 of them with a valgus malalignment of the knee joint and 21 TDs (Table 1). Solely patients with a clinical indication for a temporary hemiepiphysiodesis were included. More specifically, a pathological valgus alignment of at least one knee (38 patients were bilaterally affected) of the lower limb based on a full-length standing anteroposterior radiograph was necessary^20,49,50^. In our hospital, the indication for a temporary hemiepiphysiodesis is given when the deviation from the physiological mechanical bearing line was more than 10 mm^51^, which is approximately 3° deviation of the physiological mechanical axis angle. The static mechanical axis angle was measured as the angle formed by the line from the hip center to the knee center and the line from the knee center to the ankle center^49^. Patients did not show any other pathological disorders at the lower limb as described previously^22^. The participants for the TD study cohort were recruited from local schools. All participants and their parents were thoroughly familiarized with the gait analysis protocol. Participants and their parents gave written informed consent to participate in this study, as approved by the local ethics committee of the Goethe University Frankfurt, Germany (182/16) and in accordance with the Helsinki Declaration. The study is registered in the German Clinical Trials Register (DRKS) (number: DRKS00010296).

### Gait analysis

Kinematic data were collected barefoot at 200 Hz using an 8-camera three-dimensional motion capture system (MX 10, VICON Motion Systems, Oxford, UK). Ground reaction forces were recorded synchronously at 1000 Hz using two force plates (Advanced Mechanical Technology, Inc., Watertown, MA, USA) situated at the mid-point of the 15 m long level walkway. When analyzing frontal and transverse plane gait data, a custom made lower body protocol was used for improvement of the reliability and accuracy described in a previous study^52^. In addition to the standardized Plug-in-Gait marker set^53^, reflective markers were attached on the medial malleolus, medial femoral condyle and greater trochanter. The statically measured midpoints between the medial and lateral malleolus and condyle markers defined the centers of rotation of the ankle and knee joints^52^. The center of the hip joint was calculated with a standardized geometrical prediction method using regression Eq. (7) which is common in the clinical gait community^54^. During the static upright standing trial, participants stood barefoot, feet shoulder width apart, knees fully extended, in a forward knee position with the patella centered over the femoral condyles to control for any foot rotation effects^55^. Three to five dynamic trials with a clear foot-force plate contact were selected for further processing.

### Musculoskeletal modeling

OpenSim (4.1) was used for musculoskeletal modeling of joint angles, joint moments, muscle activations, and forces and joint contact forces^56^. Input from marker positions and ground reaction forces were prepared with the MOtoNMS toolbox (version 3) in MATLAB (2020b, The MathWorks, Inc., Natick, MA, USA) for usage in OpenSim^57^. Ankle and knee joint centers were calculated in MOtoNMS. The joint centers were the midpoints between the medial and lateral malleolus and femoral condyle markers. Force data were filtered with a zero-lag low-pass Butterworth filter and a cut-off frequency of 10 Hz. An OpenSim model^4,58^ with 23 degrees of freedom was used: six degrees of freedom for the pelvis relative to the ground frame, three for the lumbosacral joint, three for the hip joint, two for the knee joint, one for the ankle joint and one for the subtalar joint. The knee joint had sagittal and frontal-plane rotational degrees of freedom, and medial and lateral contact forces were resolved using a multi-compartment knee model^5,59^. The model was actuated by 80 muscle–tendon actuators^4,60^ and passive muscle force–length curves were calibrated using experimental data^58,61^.

The generic musculoskeletal model was linearly scaled based on marker positions and participant anthropometrics. Models were further personalized by adjusting the neutral frontal-plane alignment with the mechanical axis angle measured from X-Ray images^5^. X-rays were not available for the TD group, and the mechanical axis angle was calculated using a static gait analysis trial.^22,62^. It has been shown that this non-invasive marker-based approach correlated well with the determination of lower limb alignment in the frontal plane using radiographs in young patients with varus or valgus malalignment^62^. Inverse kinematics and inverse dynamics were calculated with the standard OpenSim processing pipelines. A static optimization implementation that incorporates tendon compliance and passive muscle forces was used to solve for muscle activations, with a cost function that minimized the sum of squared muscle activation^58^. Knee contact forces were computed and are reported as the reaction force in the medial and lateral compartments of the knee in the direction of the long axis of the tibia. All calculations were performed in MATLAB. Kinematic and kinetic parameters were segmented by gait cycle. External KJMs were normalized by body mass and KCFs were normalized by body weight (BW).

### Statistical analysis

The anthropometrics (age, body height, body mass, body mass index and the mechanical axis angle) and the walking speed of the patient group and the TD group were tested for normality using a Shapiro–Wilk test. Differences between patients and TDs of normal distributed data were compared with an independent *t*-test and non-normal distributed data with a Mann–Whitney-*U*-test (SPSS, 26, IBM Corporation, New York, NY, USA). The effect size *r* of the anthropometrics and the walking speed was calculated^63^. *r* > 0.1 described a small effect size, *r* > 0.3 a medium effect size and *r* > 0.5 a large effect size^64^. KJM and KCF mean curves between groups were statistically tested for normality and compared using a two-sample parametric *t*-test within statistical parametric mapping^65^ in MATLAB. Significant differences were considered when the critical threshold of *α* = 0.05 was passed for more than four successive time points, i.e. at least 4% of the gait cycle^66^. In the patient group, 38 participants were bilaterally affected by valgus malalignment. For the mechanical axis angle, walking speed, kinematic and kinetic comparisons between the study groups, only the more affected limb in regards of the mechanical axis angle was included. For the LMMs, both affected limbs were included. For TD, only one leg was randomly chosen to be included in all performed analyses.

For investigating the linear relationship between external KJMs and KCFs, maximal values in the first and second half of stance of the medKCFs and latKCFs and KAMs were detected (medKCF1, medKCF2, etc.). For KJM in the sagittal plane the maximal value in the first half and the minimal value of the second half of stance were extracted (KFM1 and KEM2). First, linear regression analyses between **one** predictor (e.g. KAM) and the response (e.g. medKCF) variable for the peaks in the first and second half of stance were performed. The detailed description of this analysis is reported in the supplementary material (paragraph “Linear regression analysis”). Next, LMMs were used to include multiple predictor variables and to account for both included limbs in bilateral affected patients as well as different numbers of trials included per participant and limb. In general, a minimum of three and a maximum of five trials per leg were included in the analysis. The joint moments were included as fixed and random effects. Categorical variables for the participants (subjVar) and for the analyzed leg (footVar) were implemented as random effects associating each trial with an ascending participant number and the analyzed foot (left: 1, right: 2). Knee flexion/extension moment peaks were included as squared parameters qKFM1 or qKEM2. The extracted KFM/KEM values showed deviations from a linear trend, which was checked visually. medKCF1/medKCF2 and latKCF1/latKCF2 were selected as response variables. In total, four LMMs were built for both study cohorts and additionally for the combined dataset. Building the LMMs for the combined dataset required an additional categorical parameter as fixed effect that accounted for the different groups (groupVar). For finding the best fitted LMM for the four response variables, backward selection of all included fixed effects (KAM, qKFM/qKEM, groupVar) was performed. This means that the parameters KAM, qKFM/qKEM, groupVar and all their possible interactions were included in the first fitted LMM. Step by step, non-significant fixed effects have been removed from the model until only significant related effects have been left. Additionally, only fixed effects that significantly improved *R*^2^ were included in the model to keep the models as small as possible. Random effects were excluded from the model when the variance was very small, as identified by visual inspection (approximately 10 times smaller than the variance of the residuals). In this study, the estimates, standard errors, *p*-values, the lower and upper bounds of the 95% confidence intervals, the adjusted *R*^2^, the root mean squared error (*RMSE*) in BW and as a percentage of the associated average KCF, and the coefficients for the linear regression equation are reported for each LMM. Adjusted *R*^2^ ≤ 0.09 were interpreted as little, 0.09 < *R*^2^ ≤ 0.25 as low, 0.25 < *R*^2^ ≤ 0.49 as moderate, 0.49 < *R*^2^ ≤ 0.81 as high, and *R*^2^ > 0.81 as very high correlations^32^. Statistical significance for all tests was set to *α* = 0.05. The prediction accuracy of the LMMs from the combined dataset were checked with a leave-one-out cross validation approach. The original LMM equations were used and step-by-step new LMMs were built without parameters from one trial. The parameters from the trial that was left out were then inserted in the equations and predicted the response variable. In the end, all deviations of the predicted values from the observed were used to evaluate the accuracy of the models by calculating the Sum of Squares Total (*SST*), Sum of Squares Regression (*SSR*), Sum of Squares Error (*SSE*), Mean Squared Error (*MSE*) and *RMSE* (see Supplementary Material). The results were also graphically displayed with a scatter plot of the observed vs. predicted values and Bland–Altman-Plots for each LMM (see Supplementary Material). The LMMs were built and the evaluation of the models was performed in MATLAB.

## Supplementary Information

Supplementary Information.

## Data Availability

The datasets generated and analyzed during the current study are available from the corresponding author on reasonable request.

## Abbreviations

BW: Body weight
KAM: Knee adduction moment
KCF: Knee joint contact force
KFM/KEM: Knee flexion/extension moment
KJM: Knee joint moment
latKCF: Lateral knee joint contact force
LMM: Linear mixed-effects model
medKCF: Medial knee joint contact force
qKFM/qKEM: Squared knee flexion/extension moment
TD: Typically developed healthy controls
