# Supplementary material for: Peak knee joint moments accurately predict medial and lateral knee contact forces in patients with valgus malalignment
Source: Sci Rep. 2023 Feb 18;13:2870. doi: 10.1038/s41598-023-30058-4 (PMC9938879; doi:10.1038/s41598-023-30058-4)

Supplementary Material

Search terms in Pubmed

- Search term 1: ((((opensim) OR (Anybody Technology)) AND ((gait analysis) OR (motion capture))) AND ("1980/01/01"[Date - Publication] : "2016/12/31"[Date - Publication])); performed on 2021/11/03
- Search term 2: ((((opensim) OR (Anybody Technology)) AND ((gait analysis) OR (motion capture))) AND ("2017/01/01"[Date - Publication] : "2021/11/03"[Date - Publication])); performed on 2021/11/03

Joint angles

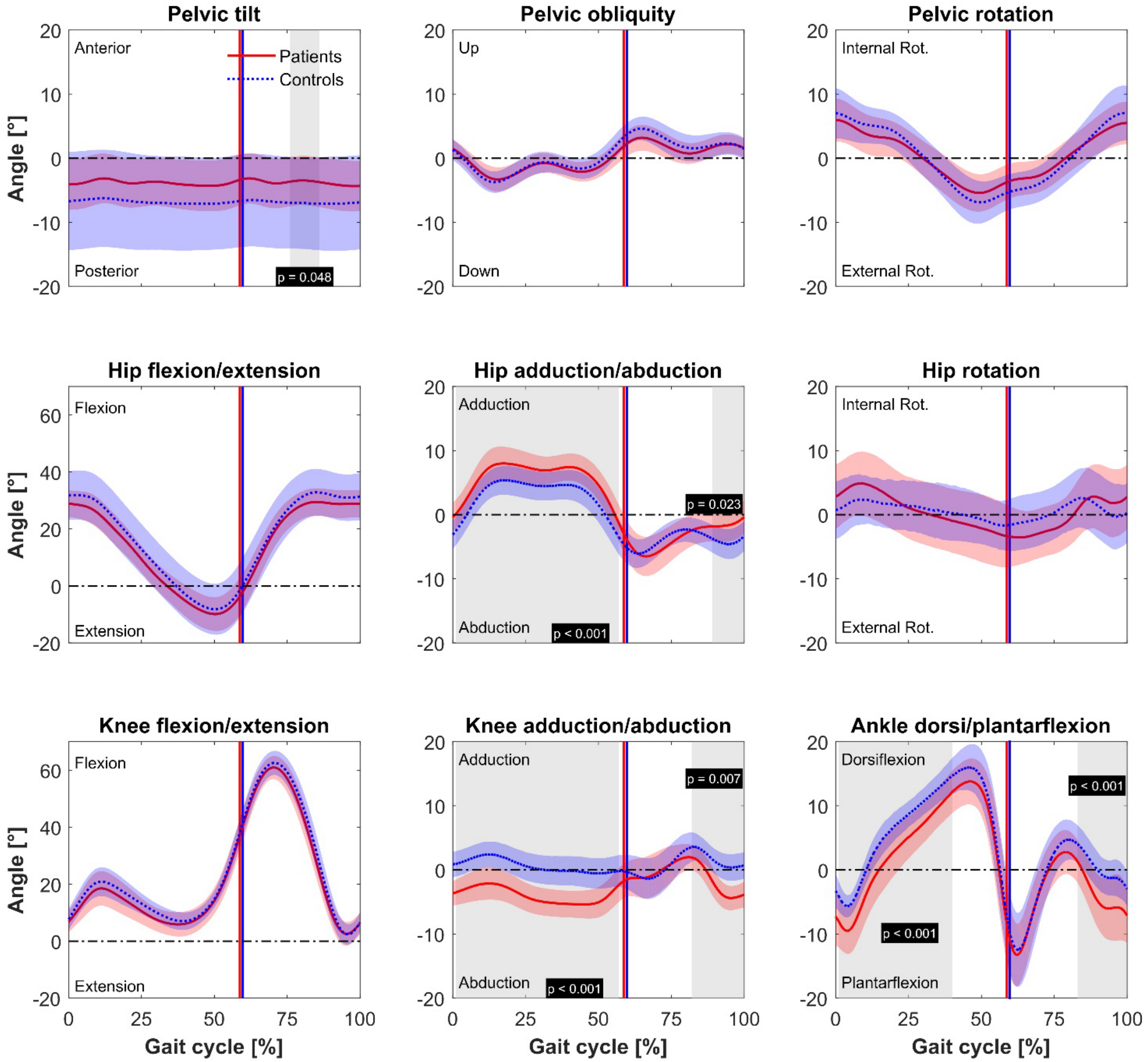

Figure 1: The mean and standard deviation of the joint angles of the pelvis, hip, knee and ankle joint between the patient (red, solid) and typically developed healthy control group (TD) (blue, dashed) are displayed. Vertical lines mark the end of the stance phase. Significant different phases ($p < 0.05$) during the gait cycle (normalized to 100 %) calculated with a statistical parametric mapping two-sample t-*t*est are highlighted with gray areas and are described with the associated *p*-value (black boxes).

Joint moments

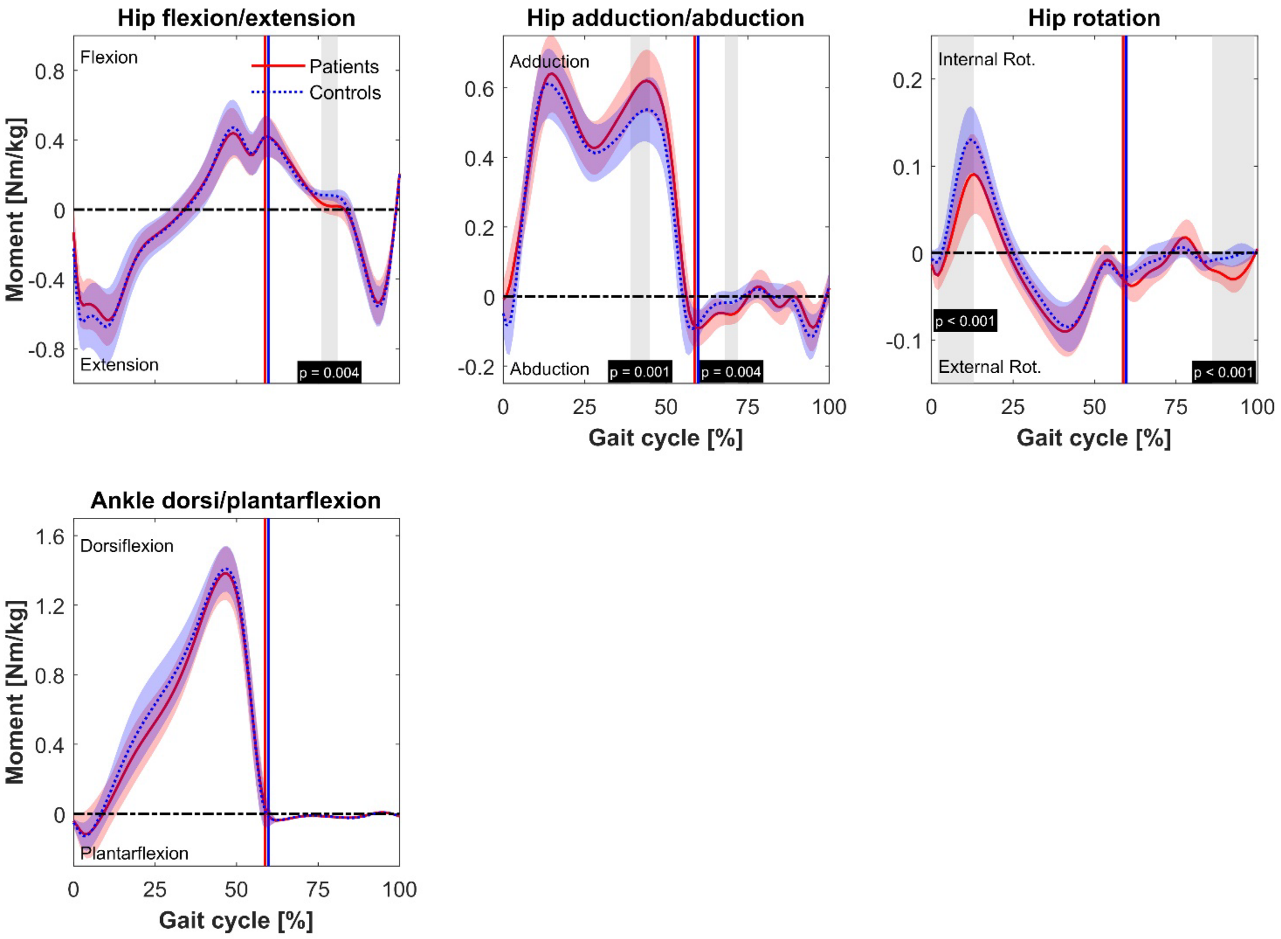

Figure 2: The mean and standard deviation of external joint moments of the hip and ankle joint between the patient (red, solid) and typically developed healthy control group (blue, dashed) are displayed. Vertical lines mark the end of the stance phase. Joint moments were normalized for body mass (unit: Nm/kg). Significant different phases ($p < 0.05$) during the gait cycle (normalized to 100 %) calculated with a statistical parametric mapping two-sample t-*t*est are highlighted with gray areas and are described with the associated *p*-value (black boxes).

Linear regression analysis

For investigating the linear relationship between **one** predictor variable (i.e., the knee flexion/extension or adduction moment) and the response variable (i.e. the medial or lateral knee joint contact force) only the more affected limb of bilateral affected patients was included in the dataset. Moreover, the mean of extracted peaks in the first and second half of stance of three trials per participant was used. In total, 50 data points per parameter for the patient group and 21 for the typically developed healthy controls group were included in the analysis. Adjusted R^2 <= 0.09 were interpreted as little, 0.09 < R^2 <= 0.25 as low, 0.25 < R^2 <= 0.49 as moderate, 0.49 < R^2 <= 0.81 as high, and R^2 > 0.81 as very high correlations adapted from Hinkle, et al. [1]. MATLAB (2020b, The MathWorks, Inc., Natick, MA, USA) was used to determine the linear relation between the predictor and response variable.Table 1: Linear regression analysis between individual predictor variables (external knee flexion/extension or adduction moment) and the response variable (internal medial or lateral knee joint contact force) for the patient group.

Response Variable	Predictor Variables	Estimate	Standard Error	*t*-value	*p*-value	Lower 95 % *CI*	Upper 95 % *CI*	Adj. R^2	RMSE	RMSE [%]
medKCF1	Intercept	1.405	0.101	13.928	< 0.001	1.202	1.607	0.47	0.29	14.40
	KAM1	2.582	0.389	6.635	< 0.001	1.800	3.365			
	Intercept	1.826	0.066	27.824	< 0.001	1.694	1.958	0.26	0.34	16.98
	qKFM1	1.134	0.266	4.268	< 0.001	0.600	1.668			
medKCF2	Intercept	1.710	0.135	12.645	< 0.001	1.438	1.981	0.26	0.48	21.48
	KAM2	3.454	0.803	4.300	< 0.001	1.839	5.070			
	Intercept	1.640	0.105	15.553	< 0.001	1.428	1.852	0.46	0.41	18.43
	qKEM2	4.157	0.639	6.507	< 0.001	2.873	5.442			
latKCF1	Intercept	1.716	0.119	14.462	< 0.001	1.477	1.954	0.11	0.34	24.02
	KAM1	-1.243	0.458	-2.714	0.009	-2.163	-0.322			
	Intercept	1.241	0.059	21.084	< 0.001	1.123	1.360	0.29	0.31	21.59
	qKFM1	1.080	0.238	4.531	< 0.001	0.601	1.559			
latKCF2	Intercept	2.075	0.062	33.523	< 0.001	1.951	2.200	0.68	0.22	14.34
	KAM2	-3.814	0.368	-10.369	< 0.001	-4.554	-3.075			
	Intercept	1.420	0.100	14.223	< 0.001	1.220	1.621	0.01	0.39	25.45
	qKEM2	0.710	0.605	1.173	0.247	-0.507	1.927			

CI: Confidence interval; Adj. R^2: adjusted R^2; RMSE: root mean squared error [BW]; medKCF1/medKCF2: max. value in the first/second half of stance of the medial knee joint contact force [BW]; latKCF1/latKCF2: max. value in the first/second half of stance of the lateral knee joint contact force [BW]; KAM1/KAM2: max. value in the first/second half of stance of the external knee adduction moment [Nm/kg]; qKFM1/qKEM2: squared maximal/minimal value in the first/second half of stance of the external knee flexion/extension moment ([Nm/kg]).

Table 2: Linear regression analysis between individual predictor variables (external knee flexion/extension or adduction moment) and the response variable (internal medial or lateral knee joint contact force) for the typically developed healthy control group.

Response Variable	Predictor Variables	Estimate	Standard Error	*t*-value	*p*-value	Lower 95 % *CI*	Upper 95 % *CI*	Adj. R^2	RMSE	RMSE [%]
medKCF1	Intercept	1.498	0.361	4.147	0.001	0.742	2.255	0.27	0.38	15.30
	KAM1	2.772	0.960	2.887	0.009	0.762	4.782			
	Intercept	2.071	0.158	13.080	< 0.001	1.739	2.402	0.32	0.37	14.71
	KFM1	1.059	0.326	3.248	0.004	0.376	1.741			
medKCF2	Intercept	1.555	0.212	7.346	< 0.001	1.112	1.998	0.59	0.37	14.15
	KAM2	4.231	0.782	5.409	< 0.001	2.594	5.869			
	Intercept	1.828	0.223	8.193	< 0.001	1.361	2.295	0.41	0.44	16.82
	qKEM2	5.470	1.404	3.895	0.001	2.531	8.410			
latKCF1	Intercept	1.214	0.333	3.651	0.002	0.518	1.910	-0.05	0.35	28.13
	KAM1	0.119	0.884	0.135	0.894	-1.731	1.969			
	Intercept	1.063	0.089	11.952	< 0.001	0.877	1.249	0.30	0.29	22.95
	qKFM1	0.792	0.256	3.096	0.006	0.256	1.327			
latKCF2	Intercept	1.761	0.138	12.730	< 0.001	1.471	2.050	0.48	0.24	20.21
	KAM2	-2.260	0.511	-4.420	< 0.001	-3.330	-1.190			

CI: Confidence interval; Adj. R^2: adjusted R^2; RMSE: root mean squared error [BW]; medKCF1/medKCF2: max. value in the first/second half of stance of the medial knee joint contact force [BW]; latKCF1/latKCF2: max. value in the first/second half of stance of the lateral knee joint contact force [BW]; KAM1/KAM2: max. value in the first/second half of stance of the external knee adduction moment [Nm/kg]; qKFM1/qKEM2: squared maximal/minimal value in the first/second half of stance of the external knee flexion/extension moment ([Nm/kg]).

[1] Hinkle, D. E., Jurs, S. G. & Wiersma, W. *Applied Statistics for the Behavioral Sciences*. 2 edn, (Houghton Mifflin, 1988).

Equations of the linear mixed-effects models for the patient group

$$medKCF1 = 1.411 + 2.187 \times KAM1 + 0.551 \times qKFM1 + (1 + KAM1 + qKFM1|subjVar) + (1|subjVar:footVar) \quad (1)$$

$$medKCF2 = 1.202 + 3.012 \times KAM2 + 4.158 \times qKEM2 + (1 + KAM2 + qKEM2|subjVar) + (1|subjVar:footVar) \quad (2)$$

$$latKCF1 = 1.544 + (-1.559) \times KAM1 + 1.498 \times qKFM1 + (1 + KAM1 + qKFM1|subjVar) + (1|subjVar:footVar) \quad (3)$$

$$latKCF2 = 1.754 + (-2.176) \times KAM2 + 0.454 \times qKEM2 + (1 + KAM2 + qKEM2|subjVar) + (1|subjVar:footVar) \quad (4)$$

Equations of the linear mixed-effects models for the typically developed control group

$$medKCF1 = 1.322 + 2.466 \times KAM1 + 0.744 \times KFM1 + (1 + KAM1 + KFM1|subjVar) \quad (5)$$

$$medKCF2 = 1.289 + 2.902 \times KAM2 + 4.133 \times qKEM2 + (1 + KAM2 + qKEM2|subjVar) \quad (6)$$

$$latKCF1 = 1.390 + (-1.112) \times KAM1 + 0.976 \times qKFM1 + (1 + KAM1 + qKFM1|subjVar) + (1|subjVar:footVar) \quad (7)$$

$$latKCF2 = 1.726 + (-2.160) \times KAM2 + (1 + KAM2|subjVar) \quad (7)$$

Equations used for calculating the statistical outcome parameters of the leave one out cross-validation:

Equation 8: Sum of Squares Total (SST) – The sum of squared differences between observed data points (y_i) and the mean of the response variable (y).

$$SST = \Sigma(y_i - y)^2$$

Equation 9: Sum of Squares Regression (SSR) – The sum of squared differences between predicted data points ($\hat{y}_i$) and the mean of the response variable (y).

$$SSR = \Sigma(\hat{y}_i - y)^2$$

Equation 10: Sum of Squares Error (SSE) – The sum of squared differences between predicted ($\hat{y}_i$) and observed data points (y_i).

$$SSE = \Sigma(\hat{y}_i - y_i)^2$$

Equation 11: Mean Squared Error – the average squared difference between predicted ($\hat{y}_i$) and observed data points (y_i).

$$MSE = \frac{\Sigma(\hat{y}_i - y_i)^2}{n}$$

Equation 12: Root Mean Squared Error – Square root of the Mean Squared Error.

$$RMSE = \sqrt{MSE}$$

Equation 13: Square of the correlation (R^2) – The ratio of the sum of squares of the regression (SSR) and the total sum of squares (SST)

$$R^2 = \frac{SSR}{SST}$$

Graphical visualization of the results

Medial knee contact force in the first half of stance

Scatter plot of observed vs. predicted parameters

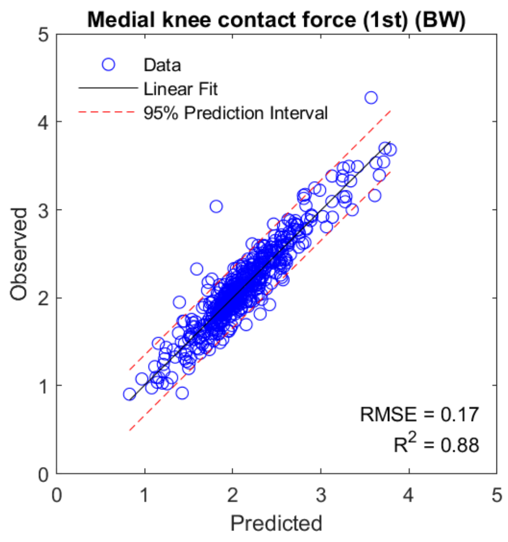

Bland-Altman Plot

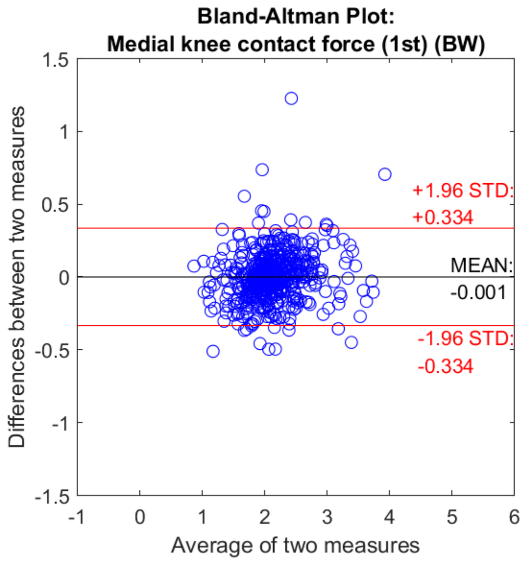

Medial knee contact force in the second half of stance

Scatter plot of observed vs. predicted parameters

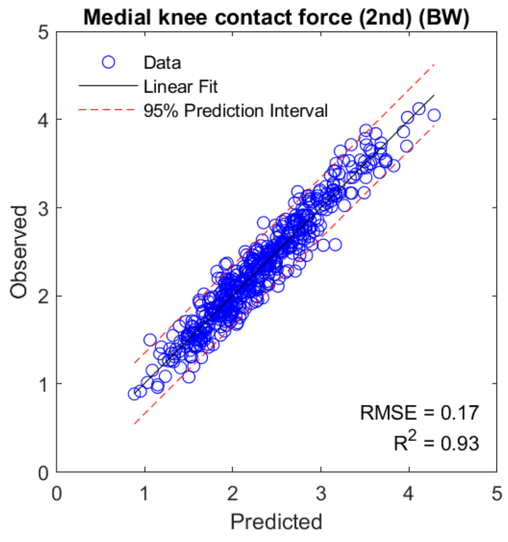

Bland-Altman Plot

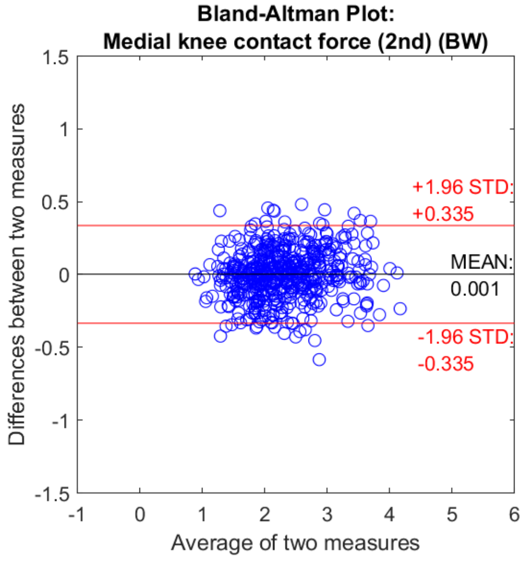

Lateral knee contact force in the first half of stance

Scatter plot of observed vs. predicted parameters

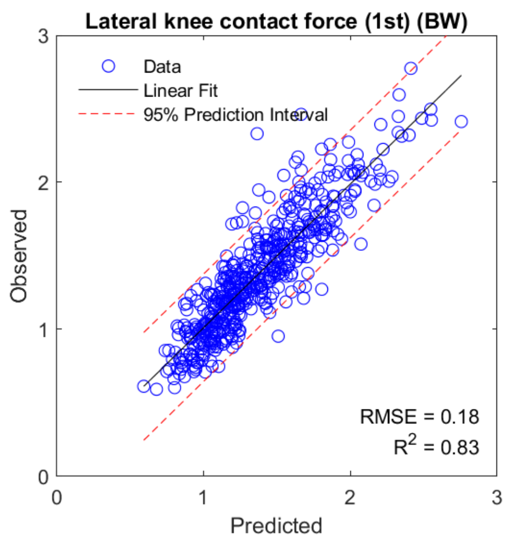

Bland-Altman Plot

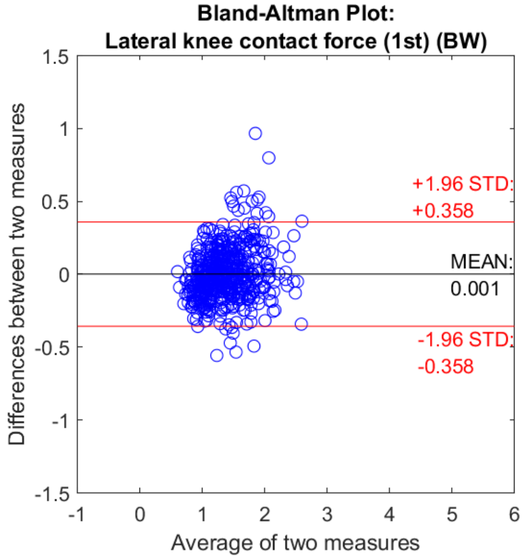

Lateral knee contact force in the second half of stance

Scatter plot of observed vs. predicted parameters

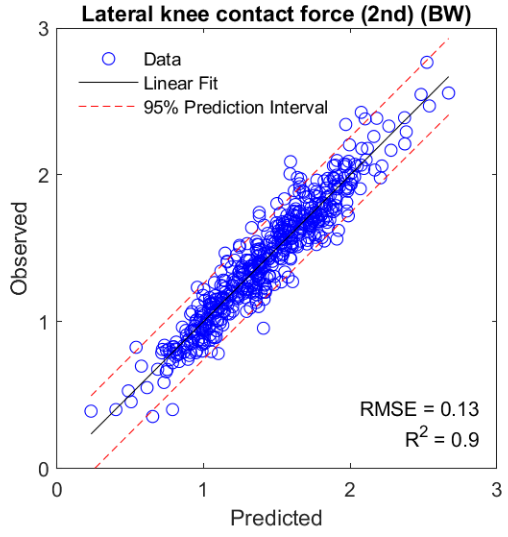

Bland-Altman Plot

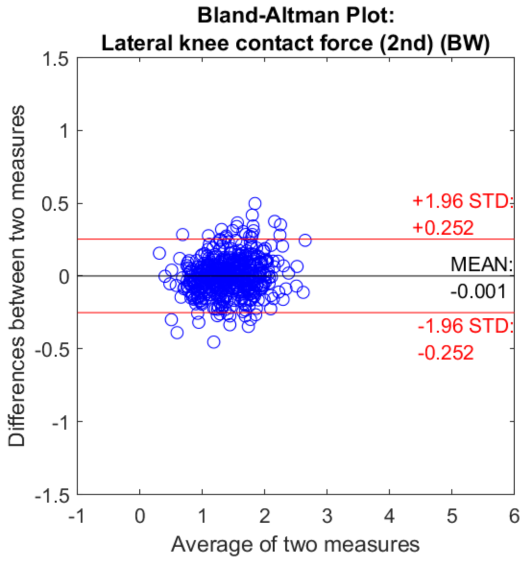

Supplement: Supplementary file 1 — Supplementary Information. [file 41598_2023_30058_MOESM1_ESM.pdf]
